# Coordination of Surface‐Induced Reaction and Intercalation: Toward a High‐Performance Carbon Anode for Sodium‐Ion Batteries

**DOI:** 10.1002/advs.201600500

**Published:** 2017-03-03

**Authors:** Weimin Chen, Chaoji Chen, Xiaoqin Xiong, Pei Hu, Zhangxiang Hao, Yunhui Huang

**Affiliations:** ^1^ Key Laboratory for Green Chemical Process of Ministry of EducationSchool of Chemical Engineering and PharmacyWuhan Institute of TechnologyWuhan430205China; ^2^ State Key Laboratory of Materials Processing and Die and Mould TechnologySchool of Materials Science and EngineeringHuazhong University of Science and TechnologyWuhan430074China

**Keywords:** carbon anodes, diffusion‐controlled intercalation, oxygen functional groups, sodium‐ion batteries, surface‐induced reaction

## Abstract

**Oxygen‐rich carbon material** is successfully fabricated from a porous carbon and evaluated as anode for sodium‐ion battery. With the strategy of optimal combination of fast surface redox reaction and reversible intercalation, the oxygen‐rich carbon anode exhibits a large reversible capacity (447 mAh g^−1^ at 0.2 A g^−1^), high rate capability (172 mAh g^−1^ at 20 A g^−1^), and excellent cycling stability.

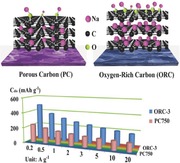

Lithium‐ion batteries (LIBs) have achieved tremendous commercial development over the last three decades because of the growing market for portable electronics and electric vehicles.^1^, ^2^, ^3^, ^4^ However, the insufficiency of lithium resource and the growing cost present unavoidable challenge to the LIB industry. To date, intensive efforts have been devoted to exploring cost‐effective rechargeable battery systems beyond LIBs.^5^, ^6^ Sodium‐ion batteries (SIBs) have been regarded as one of the most promising candidates owing to the abundant natural resources and the similar chemical properties of sodium and lithium.^7^, ^8^, ^9^ However, many electrode materials used for LIBs show very limited activity toward Na^+^ insertion because Na^+^ ion has larger ionic radius than Li^+^ and the diffusion kinetics of Na^+^ is much more sluggish. Meanwhile, a larger structural change occurs during Na^+^ insertion/deinsertion, leading to low capacity and poor cyclability. To address these concerns, a number of materials with large interstitial space in the crystallographic structure have been investigated as promising cathodes for SIBs,^10^, ^11^, ^12^ but a few have been devoted to the anode materials.

Fortunately, some materials used as SIB anodes, such as carbonaceous materials,^13^, ^14^, ^15^ sodium alloys,^16^, ^17^ Ti‐based intercalation compounds,^18^, ^19^ and metal chalcogenide materials,^20^, ^21^ have shown acceptable capacity and cyclability. However, the large expansion of sodium alloys during sodiation and the relatively low capacity of Ti‐based compounds definitely obstruct their practical applications. Therefore, carbonaceous materials, especially the low crystalline hard carbons, are proposed to be the ideal option for SIBs.^22^, ^23^ Various nanosized carbon materials with reversible capacities up to about 300 mAh g^−1^ have been investigated.^24^, ^25^, ^26^ Nevertheless, small particle size or porous structure always leads to low initial Coulombic efficiency (CE) because of the large irreversible capacity induced by high specific surface area. Recently, many studies have been conducted to enhance the Na^+^ storage performance by doping heteroatoms such as N, B, S, and P.^27^, ^28^, ^29^, ^30^ Due to the expanded interlayer distance derived by the heteroatoms, the insertion and extraction of Na^+^ between the carbon layers can be improved. Although some achievement has been obtained in the heteroatom‐doped carbon anodes, the capacity and high‐rate capability are not satisfactory because of the large radius of Na^+^ and instinct sluggish kinetics, which still hinder the Na^+^ transport in bulk materials. Additionally, the cycle life is insufficient for the practical application in SIBs.

Since the insertion and extraction of Na ions are limited in the bulk materials, it is urgent to explore new mechanisms in addition to the intercalation‐based Na^+^ storage to enhance the electrochemical performance in carbon anode. The surface‐induced Na^+^ storage mechanism based on the redox reaction between Na ions and surface oxygen functional groups, especially carbonyl groups, is proven to be an effective strategy to enhance the specific capacity and cycling stability.^31^, ^32^ On the basis of the reversible reaction between Na^+^ and carbonyl group (−C=O + Na^+^ + e^−^ ↔ −C—O—Na), many organic cathodes have shown good Na^+^ storage capacity.^33^, ^34^ Liu and co‐workers first reported carbon foams with O‐based functional groups on the surface as cathode for SIBs, which delivered a capacity of 152 mAh g^−1^ at a current density of 0.1 A g^−1^ and a capacity retention of 90% for over 1600 cycles.^31^ The introduction of oxygen functional groups to carbon structure can provide many surface reaction sites and defects for accommodation of Na ions, leading to enhanced Na storage capability and cycling life. The enhancement is believed to come from the fast Na^+^ kinetics because the surface reaction is much faster than the bulk intercalation process. Meanwhile, no obvious electrode structural transformation is observed during the surface reaction, and hence an excellent cyclability is also achieved. However, it is almost impossible to achieve a high capacity if the Na^+^ storage excessively depends on the surface‐induced mechanism while the intercalation‐based reaction is not fully taken use of,^29^ because the expanded interlayer distance can also act as accommodation for the inserted Na^+^ to contribute the capacity. Therefore, if we combine the fast surface redox reaction with reversible intercalation, an ideal electrode material with superior Na^+^ storage performance will be attained. To validate this, we fabricated four kinds of oxygen‐rich carbon (denoted as ORC) materials via two‐step pyrolytic and hydrothermal synthesis methods, and evaluated them as SIB anodes to see the Na^+^ storage behaviors and mechanisms. Benefitting from the optimum combination of surface reaction and intercalation, the optimized ORC anode delivers a reversible capacity as high as 447 mAh g^−1^ at 0.2 A g^−1^ accompanied with excellent rate capability and cyclability.

The precursor for ORC, porous carbon was synthesized by annealing potassium citrate (K_3_C_6_H_5_O_7_⋅H_2_O) at 750 °C for 1 h under an argon atmosphere (denoted as PC750). Then, the as‐prepared PC750 was functionalized by dilute nitric acid to form O‐based functional groups on the surface of carbon at 90 °C for 1.5, 3, 6, and 12 h (denoted as ORC‐1.5, ORC‐3, ORC‐6, and ORC‐12, respectively), as illustrated in **Figure** **1** (more experimental details are provided in the “Experimental Section”). The morphologies of PC750 and ORC materials were characterized by scanning electron microscopy (SEM), energy dispersive X‐ray element mapping, and transmission electron microscopy (TEM). Obviously, PC750 (Figure 1b) exhibits a porous morphology, which is well composed of fully interconnected and curled carbon nanosheets with a thickness of 10–20 nm (Figure 1b, inset). The elemental mappings show that C and O are uniformly distributed in the sample. In the formation of porous structure, the potassium compounds play a crucial role. At a relatively low temperature (<650 °C), potassium carbonate is formed, while at a higher temperature, the potassium carbonate decomposes (K_2_CO_3_ → K_2_O + CO_2_). Finally, the CO_2_ reacts with carbon (CO_2_ + C → 2CO) to achieve the porous framework.^35^ The TEM image (Figure 1c) further reveals that the carbon nanosheets of PC750 look like very thin and nearly transparent films with some thick creases. The high‐resolution TEM (HRTEM) image (Figure 1d) indicates an amorphous carbon structure of PC750, and the few‐layer stacked graphite microcrystallites with an average interlayer distance of 0.38 nm can be observed on the edge of the nanosheet. As expected, all the ORC materials inherit the porous nanosheet‐interconnected architecture of PC750 well (**Figure** **2**; Figures S1 and S2, Supporting Information), which demonstrates there is no obvious change in the morphology and structure of ORC materials after the acid functionalization. Interestingly, the HRTEM image (Figure 2c; Figure S2, Supporting Information) displays that the graphite microcrystallites in ORC have expanded interlayer distances (0.38 nm for ORC‐1.5, 0.42 nm for ORC‐3, 0.40 nm for ORC‐6, and 0.39 nm for ORC‐12). These results demonstrate that the introduction of oxygen functional groups can enlarge the interlayer distance of carbon, which is favorable for the diffusion of Na^+^ and the electrochemical utilization of carbon. Notably, the interlayer distance of carbon decreases when the reaction time is prolonged to 6 or 12 h. This could be explained that the hydrothermal reaction facilitates the graphitization of carbon and hence leads to the shrink of carbon layers.^36^, ^37^ The porous feature of PC750 and ORC materials is further confirmed by the nitrogen sorption isotherms and pore‐size distribution results (Figure 2d; Figure S3, Supporting Information). The isotherms reveal the mesoporous nature of PC750 and the specific area is 1083 m^2^ g^−1^. The specific areas are 966, 565, 595, and 685 m^2^ g^−1^ for ORC‐1.5, ORC‐3, ORC‐6, and ORC‐12, respectively, which strongly depend on the hydrothermal reaction time. The evolution in specific area indicates that some mesopores may be broken during the process of acid treatment. Therefore, the functionalization with acid plays an important role in controlling the microstructure of the ORC materials. Figure 2e compares the powder X‐ray diffraction (XRD) patterns of PC750 and ORCs. All of them present one broad peak (002) and one very weak peak (001), indicating the amorphous nature. The (002) peaks for PC750, ORC‐1.5, ORC‐3, ORC‐6, and ORC‐12 are located at around 23.5°, 22.8°, 21.4°, 21.9°, and 22.9°, respectively. According to the Bragg's law, the interlayer distances are calculated to be 3.78, 3.89, 4.14, 4.05, and 3.88 Å, which agree with the HRTEM observations.

**Figure 1:**
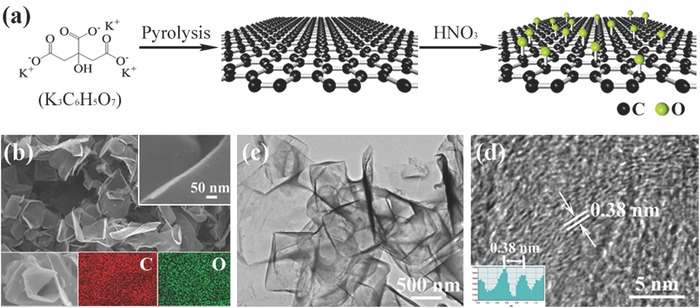
a) Schematic illustration for preparation of ORC. b) Low‐ and high‐magnification (inset) SEM images and element mapping images (inset), c) TEM image, and d) HRTEM image of PC750.

**Figure 2:**
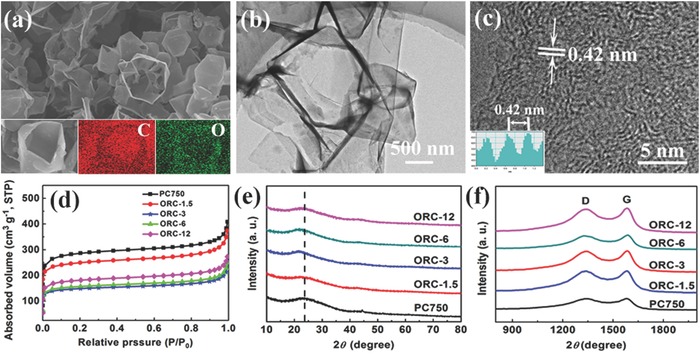
a) SEM image and element mapping images, b) TEM image, and c) HRTEM image of ORC‐3. d) Nitrogen sorption isotherms, e) XRD patterns, and f) Raman spectra of PC750 and ORC materials.

Raman spectroscopy analysis was conducted to investigate the extent of disorder structure of PC750 and ORC materials. All of the spectra in Figure 2f reveal broad disorder‐induced D‐bands (≈1340 cm^−1^) and in‐plane vibration G‐bands (≈1580 cm^−1^). The peak intensity ratio of D‐to‐G band (*I*_D_/*I*_G_) for PC750 is 0.98, while the *I*_D_/*I*_G_ values are 0.99, 1.00, 0.99, and 0.96 for ORC‐1.5, ORC‐3, ORC‐6, and ORC‐12, respectively. These data further suggest that the structural change of carbon is affected by the oxygen functional groups and the graphitization induced by the hydrothermal reaction. With increasing the hydrothermal reaction time, the structural distortion of carbon decreases while the graphitization degree increases, which is consistent with the HRTEM results.

Further structural information of PC750 and ORC materials is obtained by X‐ray photoelectron spectroscopy (XPS) and Fourier‐transform infrared (FTIR) analysis. **Figure** **3** and Figure S4 (Supporting Information) display the XPS survey spectrum and the high‐resolution XPS C 1s spectra of PC750 and four ORC materials. The peaks of oxygen functional groups can be observed from C 1s spectra of PC750 (Figure 3a) and ORC‐3 (Figure 3b). Obviously, the peaks increase after hydrothermal treatment, indicating that the oxygen functional groups increase during the acid functionalization. Based on the XPS analysis, the C/O ratios are given in Figure 3c. PC750 contains a relatively lower oxygen content (8.4 at%). After the hydrothermal reaction for 1.5 h, the oxygen content of ORC‐1.5 rapidly increases to 16.2 at%. The oxygen content further increases with the extending functionalization time. The contents are 17.5, 19.8, and 20.2 at% for ORC‐3, ORC‐6, and ORC‐12, respectively. From the high‐resolution XPS C 1s spectra, the signals of oxygen functional groups can be recognized. The O component in PC750 mainly exists in the forms of C—OH and C—O—C groups. After functionalization, the O‐based species in ORC mostly appear as C—OH, C—O—C and C=O groups. Especially, the C=O content increases remarkably, demonstrating that the hydrothermal treatment is helpful to bring the C=O groups. The functional groups are further detected by FTIR, as shown in Figure 3d. The stretching vibrations of C—OH (3450 cm^−1^), C=C (1627 cm^−1^), and C—O—C (1245 cm^−1^) are observed in all samples. Interestingly, the C=O (1720 cm^−1^) peak only appears in the ORC samples, indicating that the C=O groups are largely formed on the surface of ORC after functionalization. These results are in good accordance with XPS analysis.

**Figure 3:**
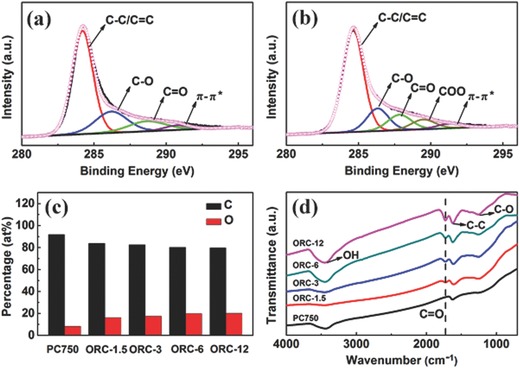
High‐resolution XPS C 1s spectra of a) PC750 and b) ORC‐3. c) C and O contents and d) FTIR spectra for PC750 and ORC samples.

The electrochemical properties of PC750 and ORC samples were investigated by cyclic voltammetry (CV) and galvanostatic charge/discharge measurement. Figure S5a,b (Supporting Information) shows the initial four CV curves of the PC750 and ORC‐3 electrodes in the range of 0.01–3 Vat a scan rate of 0.2 mV s^−1^. For PC750, a large and broad cathodic peak at about 0.5 V is observed in the first cycle and then disappears in the following cycles, which can be ascribed to the formation of solid electrolyte interphase (SEI) layer and some irreversible side reactions. The cathodic peak located at around 0.01 V is attributed to sodium‐ion insertion into carbonaceous materials.^38^ During the anodic process, no apparent peak is observed in the initial and subsequent cycles, indicating that extraction of Na ions from PC750 happens in a wide potential range.^39^ Comparing with PC750, the CV curves for ORC‐3 show two additional redox couples at 1.51/2.17 and 0.79/1.62 V, which can be ascribed to the redox reactions between Na ions and oxygen functional groups.^40^, ^41^ The similar reactions between Na ions and S were reported previously in Na–S batteries.^42^, ^43^ It indicates that the oxygen functional groups are electrochemically active to provide abundant Na storage sites, which can enhance the reversible capacity.

**Figure** **4**a displays the charge/discharge profiles for PC750 and ORC at a current density of 0.2 A g^−1^. Different from the reported typical discharge curves of hard carbon, which contain a sloping region (2–0.15 V) and a plateau region (≈0.01 V), the PC750 and ORC only show sloping region in the discharge curves, which may be resulted from the combination of the intercalation‐based Na^+^ storage and the surface‐driven Na^+^ storage based on the reactions between Na ions and oxygen functional groups.^29^ Notably, PC750 shows the lowest CE of 45.6%. With the oxygen functional groups formed on the carbon surface, the CEs of ORC samples increase gradually even to 81.5% for ORC‐12. Such a high CE has seldom been achieved in the previous reports for the carbon‐based SIB anode materials.^29^, ^44^ It suggests that the redox reactions between Na ions and oxygen functional groups are highly reversible, leading to a high CE. As shown in Figure S6 (Supporting Information), the five carbonaceous materials show good cycling stability at a low current density of 0.2 A g^−1^. The initial capacities at 0.2 A g^−1^ are 362, 447, 437, and 315 mAh g^−1^ for ORC‐1.5, ORC‐3, ORC‐6, and ORC‐12, respectively, much higher than that of PC750 (217 mAh g^−1^). During the initial several cycles, the samples show slight capacity fading, which may be ascribed to the formation of SEI and activation of electrode during the cycles. Further comparison in rate capability between PC750 and ORC is shown in Figure 4b. We can see that the ORC samples exhibit higher capacity than PC750, indicating that the introduction of oxygen functional groups can significantly improve the electrochemical performance. Obviously, ORC‐3 shows the best performance, which delivers 368, 312, 272, 245, 216, and 194 mAh g^−1^ at 0.5, 1, 2, 3, 5, and 10 A g^−1^. Even at a high current density of 20 A g^−1^, a capacity as high as 172 mAh g^−1^ is still attained, much higher than the previous reports on carbon‐based electrode (Figure S7, Supporting Information). Figure S8 (Supporting Information) shows the cycling performance at a current density of 1 A g^−1^. With activation at 0.2 A g^−1^ for initial ten cycles, the reversible capacities are kept at 148, 194, 243, 218, and 155 mAh g^−1^ for PC750, ORC‐1.5, ORC‐3, ORC‐6, and ORC‐12, respectively, after 800 cycles. As expected, the ORC samples show better cyclability PC750, and ORC‐3 presents the best cycling performance. We focus on the ORC‐3 sample. After activation at 0.2 A g^−1^ for initial ten cycles, the galvanostatic charge/discharge tests were carried out at high current densities for long cycles. After 1000 cycles, the capacity remains 225 mAh g^−1^ at 2 A g^−1^ (Figure 4c) and 164 mAh g^−1^ at 5 A g^−1^ (Figure 4d), corresponding to 0.023% and 0.031% capacity decay per cycle, respectively. In addition, the CE value retains nearly 100% during the cycling, further indicative of excellent reversibility.

**Figure 4:**
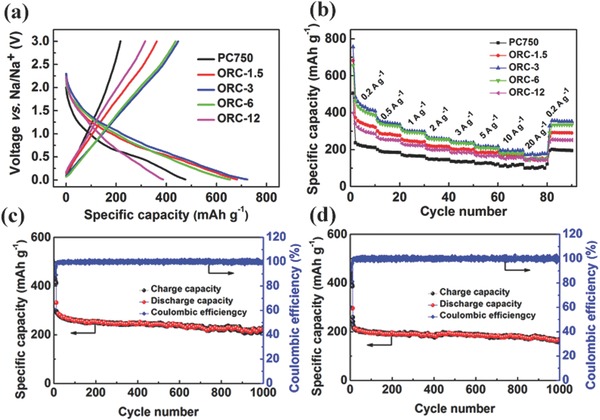
a) Charge/discharge curves at 0.2 A g^−1^ and b) rate performances at different current densities for PC750 and ORC. Cycling performance of ORC‐3 at c) 2 A g^−1^ and d) 5 A g^−1^.

To gain a better understanding of the excellent electrochemical performance for ORC, ex situ FTIR analysis was performed at the selected potentials, as indicated in the charge/discharge profile (**Figure** **5**a). As shown in Figure 5b,c, the peak at 1705 cm^−1^, ascribed to C=O groups, is gradually weakened upon discharging to 0.01 V, demonstrating that most of the C=O groups are involved in the redox reactions during the sodiation process. With discharging, two new peaks appear at 1760 and 1780 cm^−1^, indicating that some new groups are formed. After recharging to 3.0 V, the peak at 1705 cm^−1^ gradually recovers, indicating the reformation of C=O groups due to desodiation. Interestingly, the peak at 1780 cm^−1^ disappears, while the peak at 1760 cm^−1^ does not change much during the charging process. Considering the high charge/discharge reversibility of the ORC materials, the disappeared peak at 1780 cm^−1^ may be attributed to the reversibly formed sodium enolate groups (C—O—Na), while the remained peak at 1760 cm^−1^ corresponds to the formed SEI film on the surface of the electrode.^34^, ^45^ Furthermore, the ex situ FTIR spectrum of the fully charged ORC‐3 electrode after 20 cycles maintains almost the same as that in the initial cycle (Figure S9, Supporting Information). These results demonstrate that the redox reaction between Na^+^ and C=O group is highly reversible, and the SEI film is very stable, giving rise to high Coulombic efficiency, large capacity, and good cyclability. Figure S10 (Supporting Information) compares the electrochemical impedance spectra (EIS) between the PC750 and ORC‐3 cells after different cycles. The Nyquist plots for each cell show a semicircle at the medium frequencies, which corresponds to the charge transfer resistance (*R*_ct_), and a straight line at the low frequencies which is attributed to sodium ion diffusion inside the active materials. The kinetic parameters obtained from the equivalent circuit fitting are listed in Table S1 (Supporting Information). The *R*_ct_ value for ORC‐3 is 263 Ω after 50 cycles, and then reduces to 172 Ω after 200 cycles. After 300 cycles, the *R*_ct_ value is stabilized at about 220 Ω, much smaller than that of PC750, indicating that oxygen functional groups on the carbon surface facilitate the formation of stable SEI film, Na^+^‐ion insertion/extraction, and charge transfer at the electrode/electrolyte interface.

**Figure 5:**
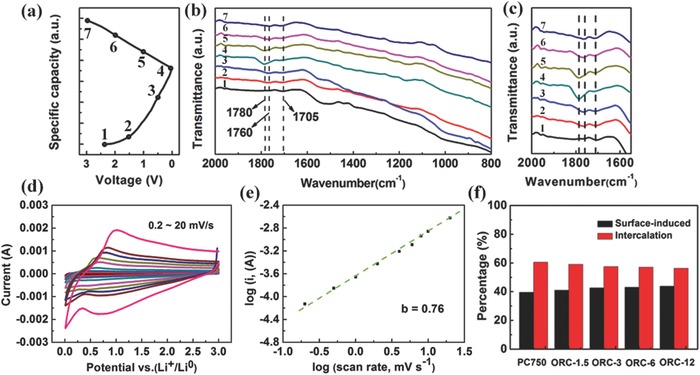
a) Charge/discharge profile of the ORC‐3 electrode during the initial cycle at 0.2 A g^−1^. b,c) Ex situ FTIR spectra of ORC‐3 at the selected potentials indicated in part (a). d) CV curves at various rates from 0.2 to 20 mV s^−1^ for ORC‐3. e) The log(ν)–log(*i*) profiles of ORC‐3. f) Capacity contributions from surface capacitance and diffusion‐controlled process for PC750 and four ORC samples.

To further investigate the Na^+^ storage mechanism for PC750 and ORCs, CV analysis was conducted at various scan rates from 0.2 to 20 mV s^−1^, as shown in Figure 5d,e and in Figure S11 (Supporting Information). The contribution from intercalation and surface capacitive reaction can be described according to the equation *i* = *av^b^*.^46^ A *b*‐value of 1 indicates that the reaction is mostly controlled by the surface capacitive process, while the *b*‐value of 0.5 suggests a diffusion‐controlled intercalation. The *b*‐values at the cathodic peaks of PC750 and four ORC samples are estimated as 0.75–0.77, demonstrating that both types of reactions contribute to the capacity. Using the method proposed by Dunn and co‐workers, the ratio of the two contributions can be quantitatively separated.^47^ Based on the *i* = *k*_1_ν +*k*_2_ν^1/2^ equation, the surface capacitive contributions are calculated to be 39.6% (PC750), 41.0% (ORC‐1.5), 42.6% (ORC‐3), 43.0% (ORC‐6), and 43.8% (ORC‐12) at 1 mV s^−1^ (Figure 5f; Figure S12, Supporting Information), indicating that the ratio of the surface capacitive contribution increases with the oxygen group content.

In general, the surface capacitive reaction is fast and reversible, but ORC‐12 with a higher contribution from the surface capacitance shows a lower capacity as compared with ORC‐3. By combining the XRD patterns and TEM images, we propose the Na^+^ storage mechanism in **Figure** **6** for PC750 and ORCs. As we know, the Na^+^ storage capacity of the carbon strongly depends on the interlayer distance and the surface functional groups. For PC750, the relatively small interlayer distance (3.78 Å) and low surface functional group content make it difficult to electrochemically intercalate and surface‐uptake Na^+^ ions, resulting in a very low total capacity. For ORC‐3, the large interlayer distance (4.14 Å) is favorable to insert/extract Na^+^ ions, the abundant surface functional groups facilitate fast surface redox reactions, leading to a superior electrochemical performance. However, the situation is different for ORC‐12, in which a large number of oxygen functional groups introduced to carbon shorten the interlayer distance due to graphitization of carbon. Although the surface redox reactions are still available, the electrochemical intercalation of Na^+^ is limited because of the insufficient interlayer distance. As a result, the electrochemical performance of ORC‐12 becomes poor. Therefore, an excellent Na^+^ storage performance cannot be attained if the capacity contribution just comes from the diffusion‐controlled intercalation or from surface‐induced reaction. An ideal electrode material for SIBs should coordinate both types of contributions, i.e., the fast surface reaction and reversible intercalation.

**Figure 6:**
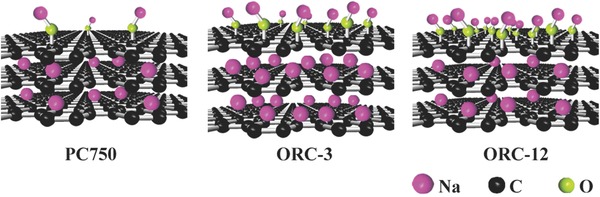
Schematic diagrams for Na storage mechanism for PC750, ORC‐3, and ORC‐12.

In summary, oxygen‐rich carbon materials were successfully fabricated from a porous carbon and evaluated as SIB anodes. The samples with different interlayer distance and oxygen content exhibit different Na^+^ storage performance. The ORC‐3 anode with larger interlayer distance and abundant surface oxygen functional groups presents the highest specific capacity and the best rate capability. The large interlayer distance can facilitate the fast insertion/extraction of Na^+^, and the surface functional groups can further provide plentiful Na^+^ storage sites. Through the proper coordination of surface redox reaction and intercalation, ORC‐3 shows a large capacity of 447 mAh g^−1^ at 0.2 A g^−1^, high rate capability (172 mAh g^−1^ at 20 A g^−1^), and excellent cycling stability. Our results not only provide a better understanding of Na^+^ storage mechanisms in carbon‐based anodes but also open up a new design strategy to develop high‐performance carbonaceous materials for SIBs.

## Experimental Section

*Materials Synthesis—Synthesis of Porous Carbon (PC750)*: Typically, 10 g potassium citrate (K_3_C_6_H_5_O_7_·H_2_O) was annealed at 750 °C for 1 h in argon with a heating ramp of 3 °C min^−1^. Then, the obtained black solid was washed with dilute HCl solution and deionized water till the filtrate became neutral. Finally, the solid was dried overnight at 80 °C in an oven, and the sample was denoted as PC750.

*Materials Synthesis—Synthesis of ORC*: ORC samples were synthesized by functionalization of PC750 using dilute HNO_3_. In brief, PC750 was dispersed in HNO_3_ solution (20%) and homogenized by ultrasound for 30 min. The resultant mixture was then transferred into a Teflon‐lined stainless steel autoclave and heated at 90 °C for 1.5, 3, 6, and 12 h (denoted as ORC‐1.5, ORC‐3, ORC‐6, and ORC‐12, respectively). Finally, the solid precipitates were washed with deionized water and dried in a vacuum at 80 °C for 12 h.

*Materials Characterization*: The morphology and microstructure were observed by SEM (FEI SIRION‐200) and TEM (JEOLJEM‐2010 (HT) electron microscope). The phase was checked by XRD (XRD‐6000, Japan with Cu Kα radiation of λ = 0.15406 nm), Raman spectra (LabRAM HR800), and FTIR (Bruker, VERTEX70). XPS measurement was performed on a VG MultiLab 2000 system with a monochromatic Al Kα X‐ray source (Thermo VG Scientific). The specific Brunauer–Emmett–Teller surface area and pore size distribution were analyzed by a Micromeritics ASPA 2020 instrument.

*Electrochemical Characterization*: Electrochemical properties of the PC750 and ORC materials were measured with 2032 coin‐type cells. Na metal was used as the counter electrode, 1 mol L^−1^ NaClO_4_ in a mixture of ethylene carbonate and propylene carbonate (1:1 by volume) as the electrolyte, and glass fiber (GF/D, Whatman) as the separator. The working electrode was made from the slurry containing PC750 or ORC (80 wt%), super P (10 wt%), and polyvinylidene fluoride (10 wt%) coated on a copper foil substrate. The galvanostatic charge/discharge tests were carried out between 0.01−3.0 V versus Na^+^/Na on a Land battery measurement system (Wuhan, China) at room temperature. CV measurement and EIS test were performed on a PARSTAT 2273 potentiostat (USA).

## Supporting information

As a service to our authors and readers, this journal provides supporting information supplied by the authors. Such materials are peer reviewed and may be re‐organized for online delivery, but are not copy‐edited or typeset. Technical support issues arising from supporting information (other than missing files) should be addressed to the authors.

Supplementary
