# Supplementary material for: Coordination of Surface‐Induced Reaction and Intercalation: Toward a High‐Performance Carbon Anode for Sodium‐Ion Batteries
Source: Adv Sci (Weinh). 2017 Mar 3;4(6):1600500. doi: 10.1002/advs.201600500 (PMC5473325; doi:10.1002/advs.201600500)
Supplement: Supplementary file 1 — Supplementary [file ADVS-4-na-s001.pdf]

Copyright WILEY-VCH Verlag GmbH & Co. KGaA, 69469 Weinheim, Germany, 2017.

ADVANCED SCIENCE
Open Access

Supporting Information

for *Adv. Sci.,* DOI: 10.1002/advs.201600500

Coordination of Surface-Induced Reaction and Intercalation: Toward a High-Performance Carbon Anode for Sodium-Ion Batteries

*Weimin Chen, Chaoji Chen, Xiaoqin Xiong, Pei Hu, Zhangxiang Hao, and Yunhui Huang**

Copyright WILEY-VCH Verlag GmbH & Co. KGaA, 69469 Weinheim, Germany, 2013.

Supporting Information

Coordination of Surface-Induced Reaction and Intercalation: Towards a High-Performance Carbon Anode for Sodium-Ion Batteries

*Weimin Chen, Chaoji Chen, Xiaoqin Xiong, Pei Hu, Zhangxiang Hao, and Yunhui Huang**

E-mail: huangyh@hust.edu.cn

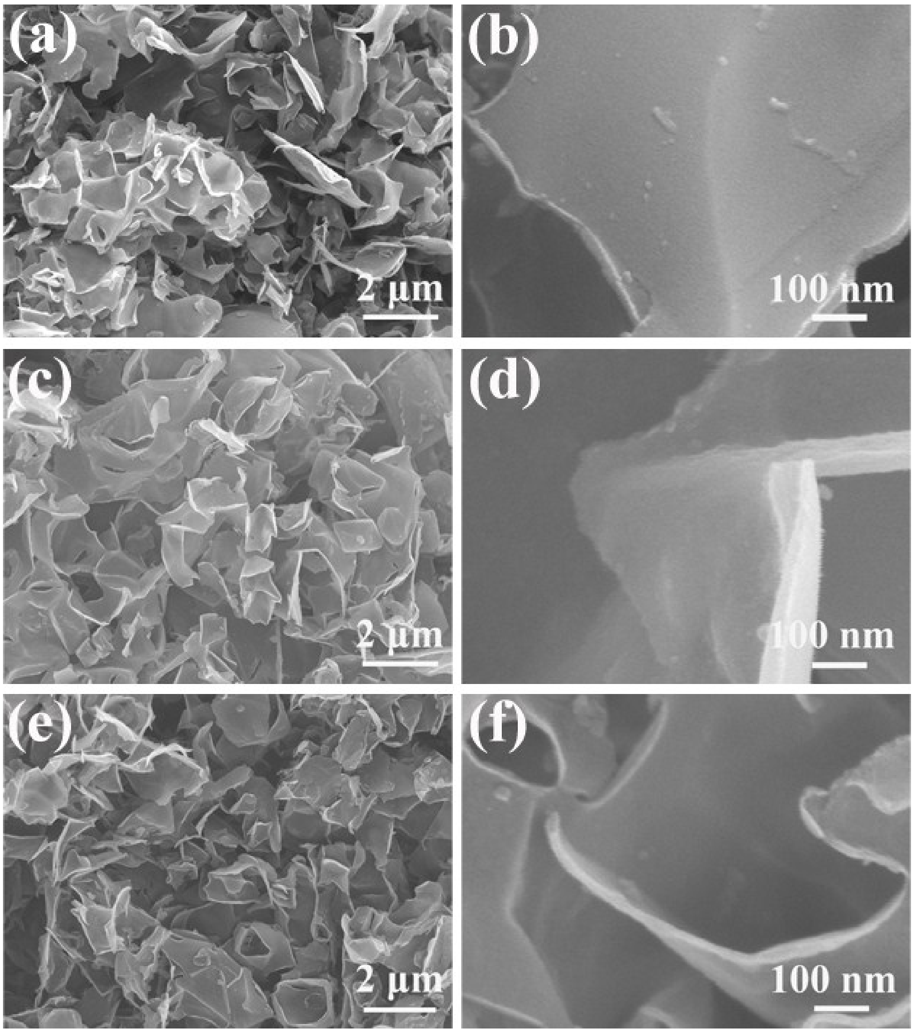

Figure S1. SEM images of (a, b) ORC-1.5, (c, d) ORC-6, and (e, f) ORC-12.

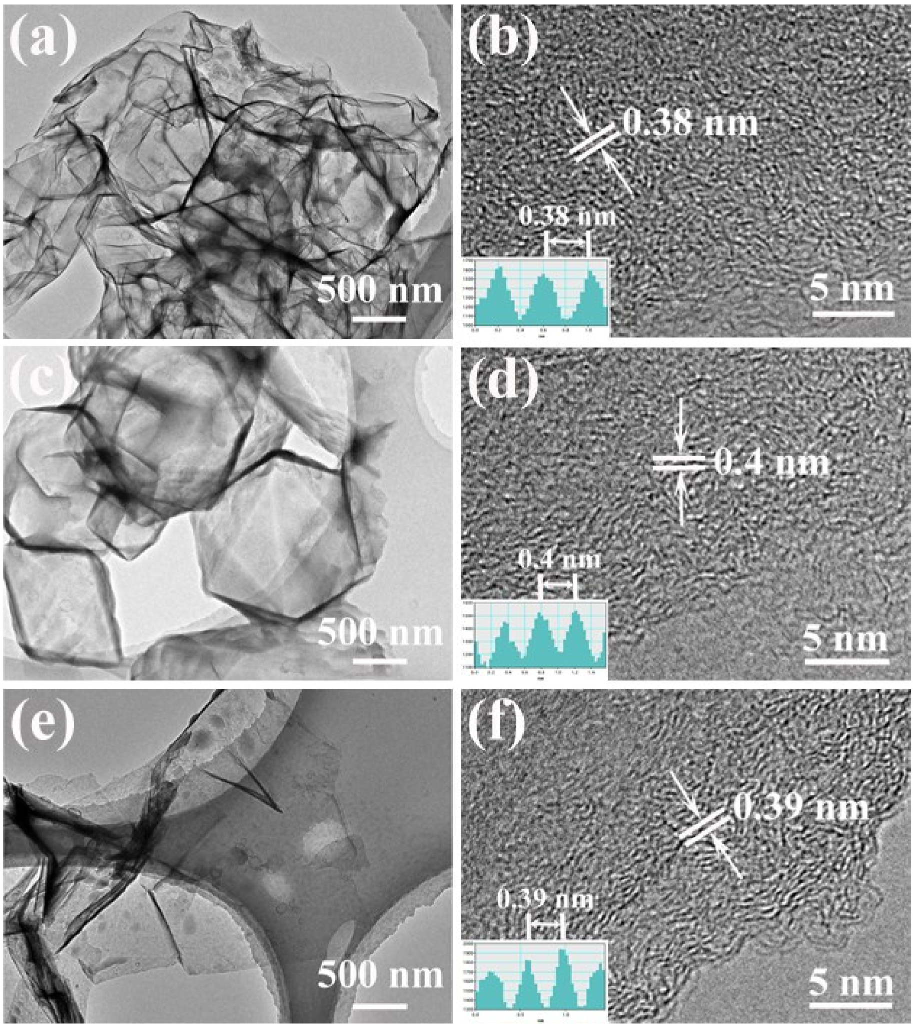

Figure S2. TEM and HRTEM images of (a, b) ORC-1.5, (c, d) ORC-6, and (e, f) ORC-12.

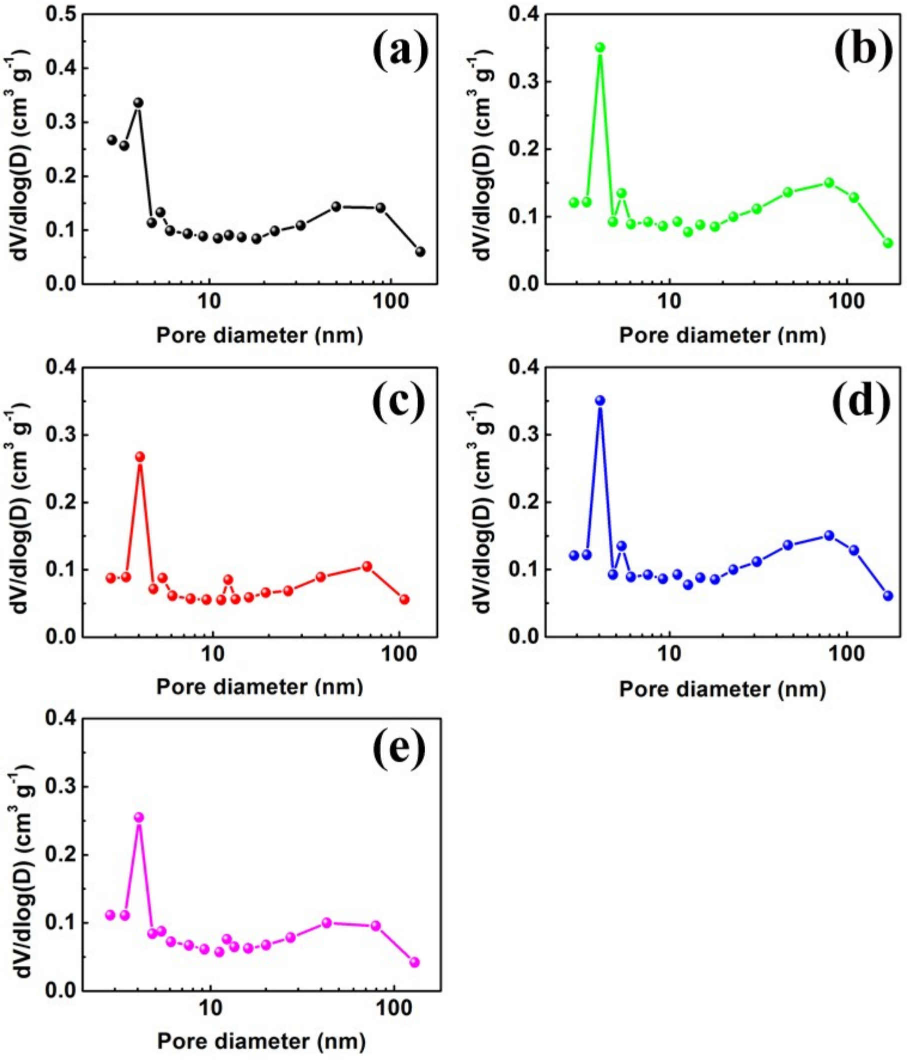

Figure S3. Pore size distribution of (a) PC750, (b) ORC-1.5, (c) ORC-3, (d) ORC-6, and (e) ORC-12.

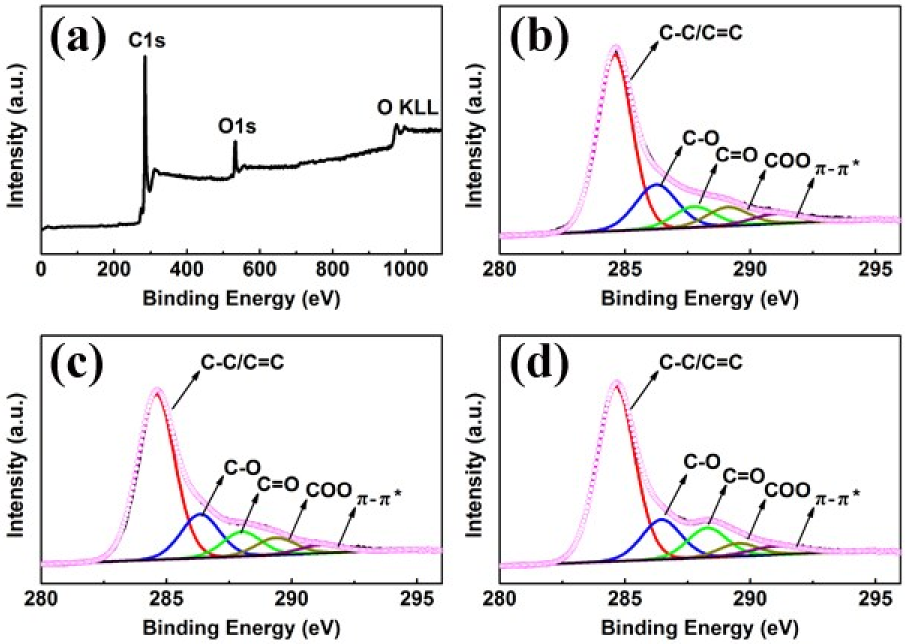

Figure S4. (a) XPS survey spectrum of PC750; high-resolution XPS C1s spectra of (b) ORC-1.5, (c) ORC-6, and (d) ORC-12.

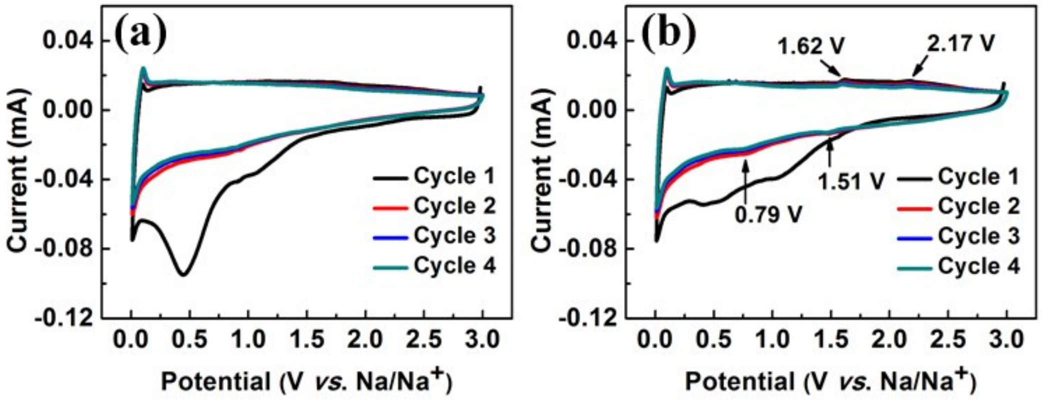

Figure S5. CV curves of (a) PC750 and (b) ORC-3.

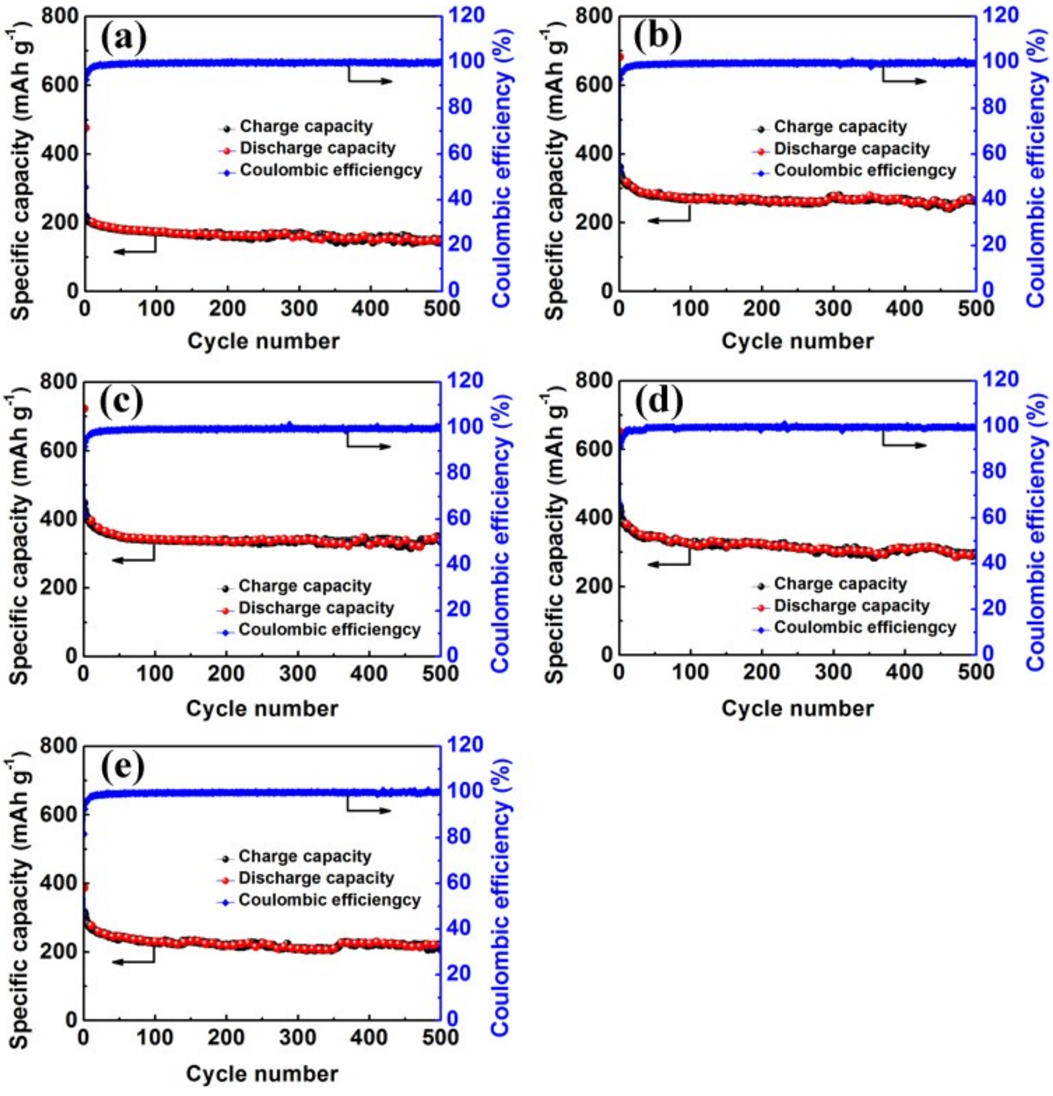

Figure S6. Cycling performances of (a) PC750, (b) ORC-1.5, (c) ORC-3, (d) ORC-6, and (e) ORC-12 at a current density of 0.2 A g^{-1}.

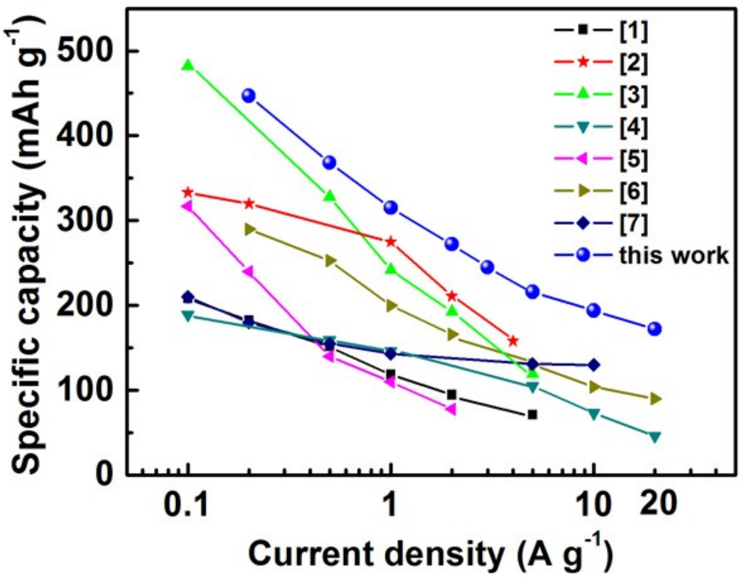

Figure S7. Rate-performance comparison between this work and previous results. In addition, the initial coulombic efficiency of the anodes are 27% at 0.05 A g^{-1} for [1], 63.2 % at 0.02 A g^{-1} for [2], 73.6% at 0.1 A g^{-1} for [3], 67.7% at 0.03 A g^{-1} for [5], 34.8% at 0.5 A g^{-1} for [6], 26.7% at 0.5 A g^{-1} for [7], and 61.8 at 0.2 A g^{-1} for this work. Compared with previously published results of carbon-based anodes, the ORC-3 electrode in this work exhibits higher coulombic efficiency and stability at high-rate capability.

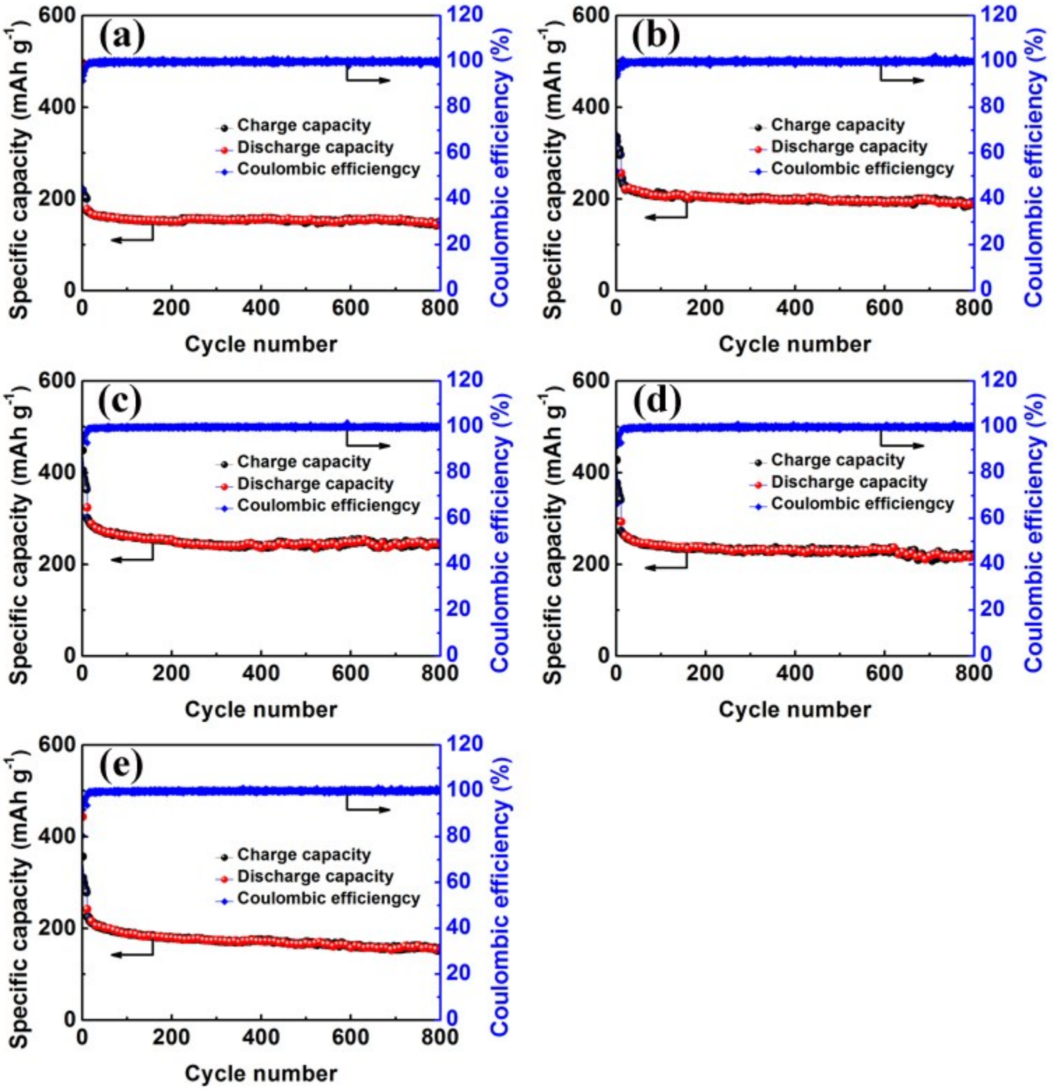

Figure S8. **C**ycling performances of (a) PC750, (b) ORC-1.5, (c) ORC-3, (d) ORC-6, and (e) ORC-12 at 1.0 A g^{-1}.

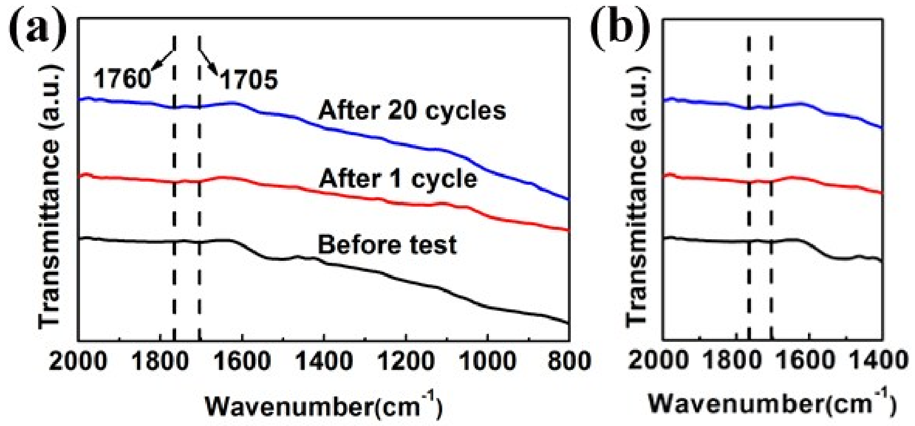

Figure S9. (a, b) *Ex-situ* FTIR spectra of the ORC-3 electrodes before and after cycling at 0.2 A g^{-1}.

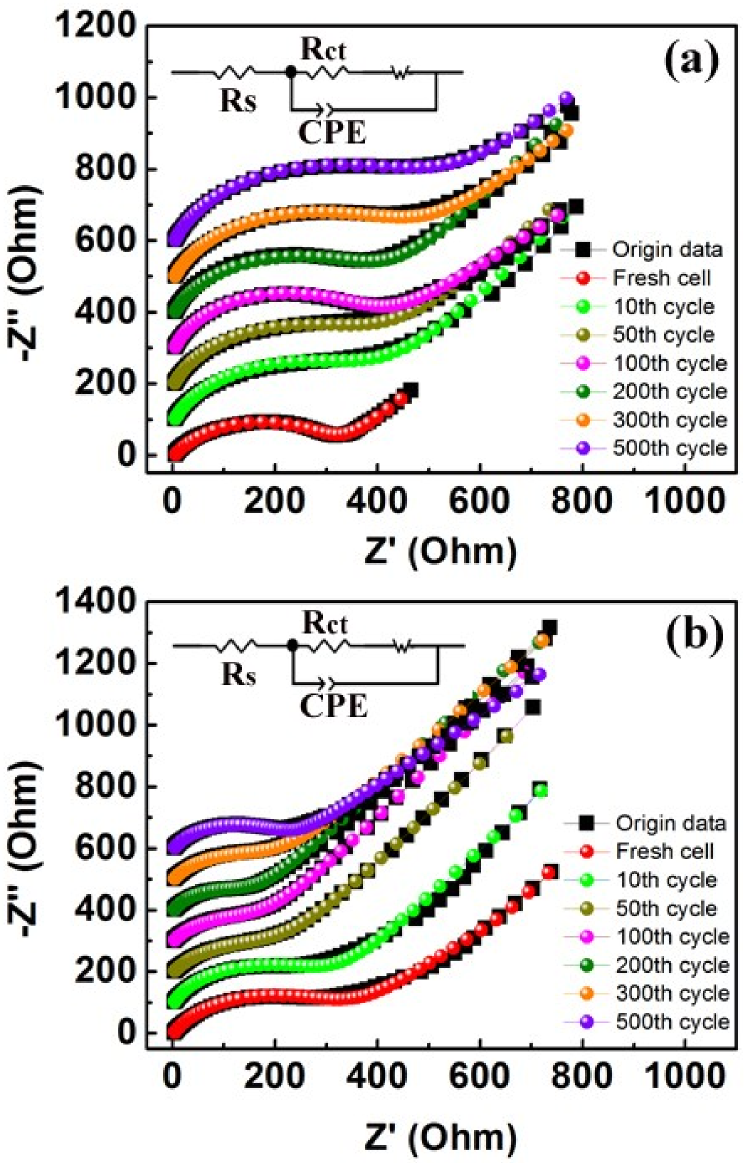

Figure S10. The Nyquist plots of (a) PC750 and (b) ORC-3 electrodes before and after different cycles at a current density of 0.2 A g^{-1} with the inset showing the equivalent circuit used for data fitting (black squares, experimental data; color spheres, fitting results).

Table S1. Simulated impendence parameters (R_s, R_{ct}) obtained from the equivalent circuit fitting of EIS tests before and after different cycles of PC750 and ORC-3 anodes at a current density of 0.2 A g^{-1}.

	PC750		ORC-3	
	R_s (Ω)	R_{ct} (Ω)	R_s (Ω)	R_{ct} (Ω)
Before test	4.8	327	3.2	324
10th cycle	4.5	425	3.4	321
50th cycle	4.2	415	3.7	263
100th cycle	4.8	385	3.5	222
200th cycle	4.8	423	3.2	172
300th cycle	4.7	474	3.3	226
500th cycle	4.8	524	3.3	226

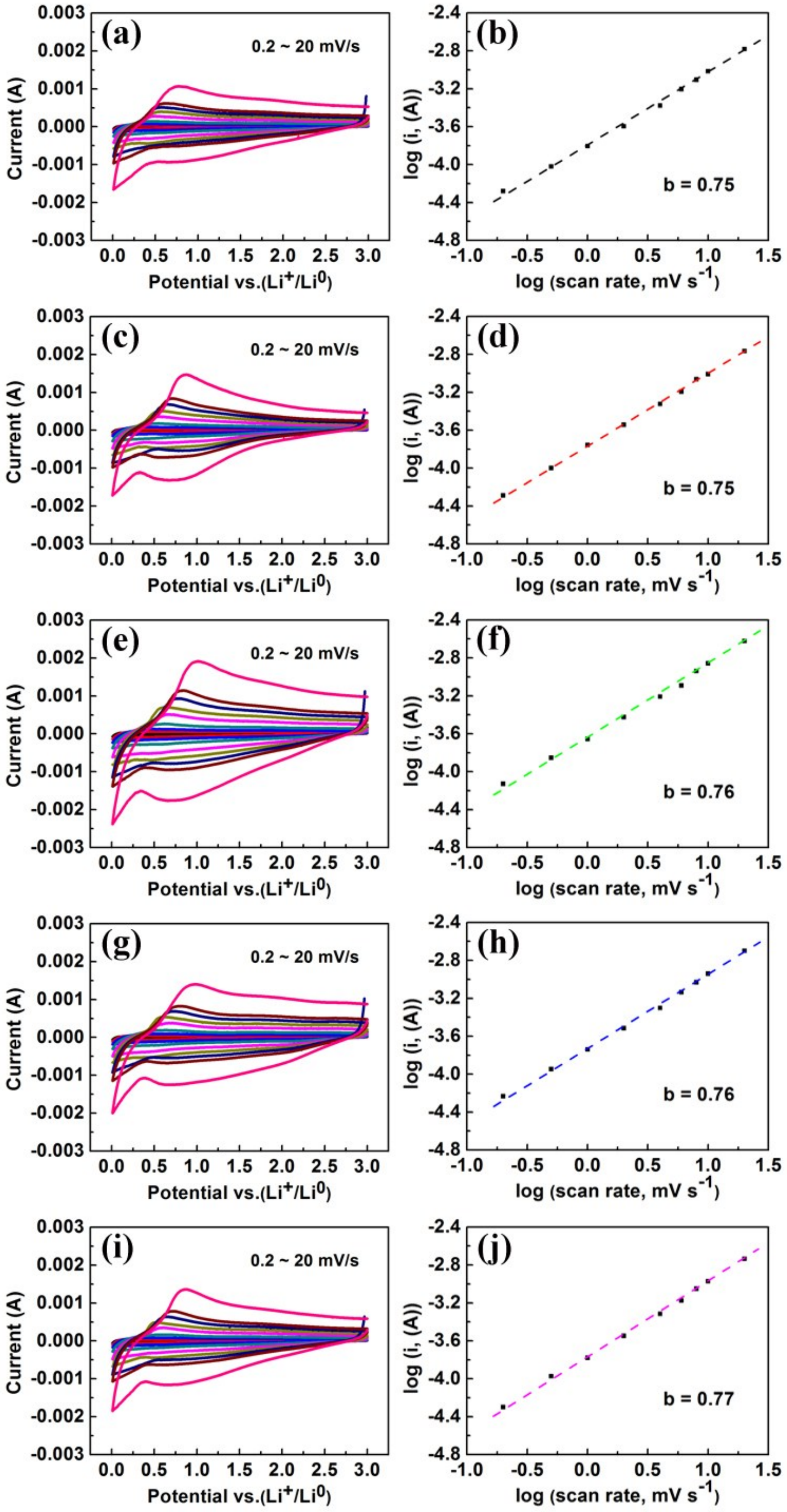

Figure S11. CV curves at various scan rates (v) from 0.2 to 20 mV s^{-1} for (a) PC750, (c) ORC-1.5, (e) ORC-3, (g) ORC-6, and (i) ORC-12. The log(v)-log(i) profiles of (b) PC750, (d) ORC-1.5, (f) ORC-3, (h) ORC-6, and (j) ORC-12.

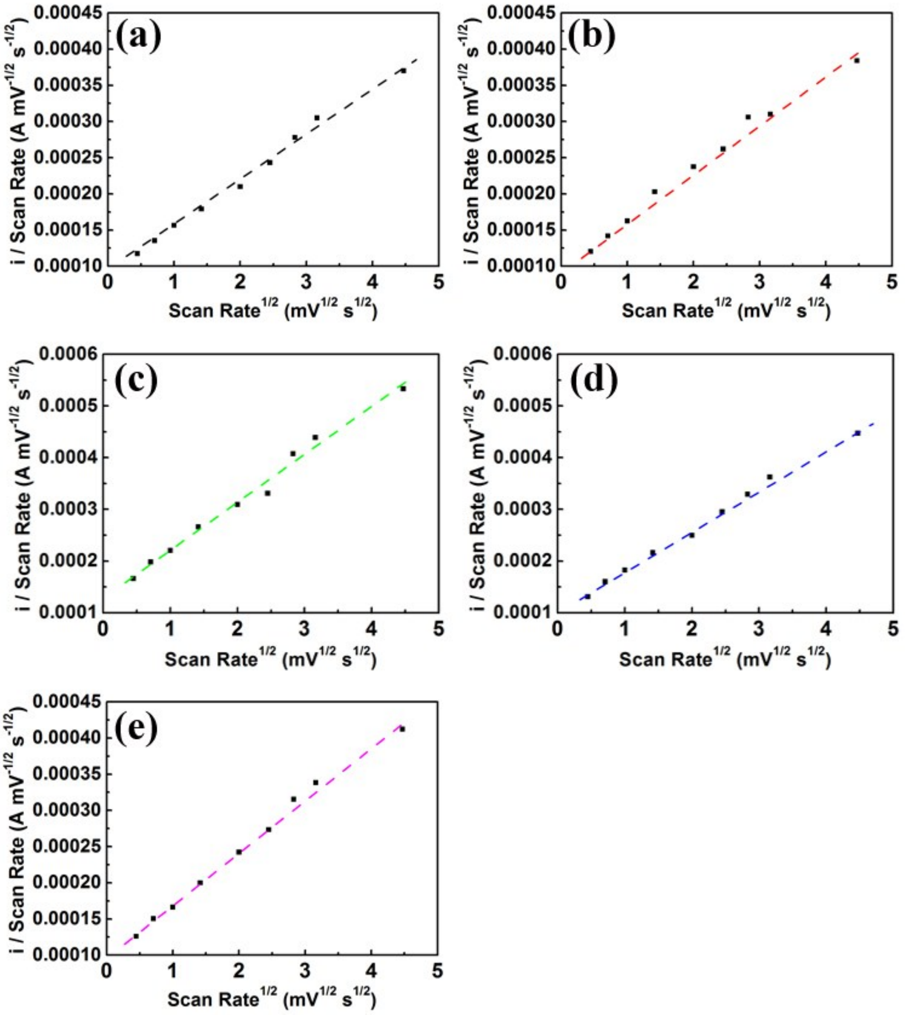

Figure S12. The plots of $i/v^{1/2}$ *vs.* $v^{1/2}$ at 1.0 mV s^{-1} of (a) PC750, (b) ORC-1.5, (c) ORC-3, (d) ORC-6, and (e) ORC-12.

References

[1] K. Hong, L. Qie, R.Zeng, Z. Yi, W. Zhang, D. Wang, W. Yin, C. Wu, Q. Fan, W. Zhang, Y. Huang, *J. Mater. Chem. A* **2014**, *2*, 12733.

[2] W. Li, M. Zhou, H. Li, K. Wang, S. Cheng, K. Jiang, *Energy Environ. Sci*. **2015**, *8*, 2916.

[3] L. Qie, W. Chen, X. Xiong, C. Hu, F. Zou, P. Hu, Y. Huang, *Adv. Sci*. **2015**, *2*, 1500195.

[4] X. Luo, C. Yang, Y. Peng, N. Pu, M. Ger, C. Hsieh, J. Chang, *J. Mater. Chem. A* **2015**, *3*, 10320.

[5] N. Sun, H. Liu, B. Xu, *J. Mater. Chem. A* **2015**, *3*, 20560.

[6] H. Hou, C. E. Banks, M. Jing, Y. Zhang, X. Ji, *Adv. Mater*. **2015**, *27*, 7861.

[7] D. Xu, C. Chen, J. Xie, B. Zhang, L. Miao, J. Cai, Y. Huang, L. Zhang, *Adv. Energy Mater*. **2016**, *6*, 1501929.
